# Development of two ultra-sensitive UHPLC–QqQ-MS/MS methods for the simultaneous determination of hydroxyzine and its active metabolite (cetirizine) in human blood: applications to real cases of forensic toxicology

**DOI:** 10.1007/s00204-024-03867-3

**Published:** 2024-09-28

**Authors:** Agnieszka Chłopaś-Konowałek, Paweł Szpot, Marcin Zawadzki, Wirginia Kukula-Koch, Ewa Dudzińska

**Affiliations:** 1 Department of Forensic Medicine, Division of Molecular Techniques, Faculty of Medicine, Wroclaw Medical University, Sklodowskiej-Curie 52, 50369 Wroclaw, Poland; 2 Department of Forensic Medicine, Faculty of Medicine, Wroclaw Medical University, 4 J. Mikulicza-Radeckiego Street, 50345 Wroclaw, Poland; 3 Department of Social Sciences and Infectious Diseases, Faculty of Medicine, Wroclaw University of Science and Technology, 27 Wybrzeze Wyspianskiego Street, 50370 Wroclaw, Poland; 4 Department of Pharmacognosy with Medicinal Plants Garden, Medical University of Lublin, 1 Chodzki Str., 20‐093 Lublin, Poland; 5 Department of Dietetics and Nutrition Education, Medical University of Lublin, 7 Chodzki Str., 20-093 Lublin, Poland

**Keywords:** Antihistamine drug, Hydroxyzine, Cetirizine, QqQ-MS/MS, Forensic samples

## Abstract

Both postmortem toxicological and medical-forensic examinations are very important in the case of analyzing various types of chemical substances. Hydroxyzine (HZ) is a first-generation antihistamine drug with a sedative effect that disrupts cognitive function and affects the ability to drive motor vehicles. Enzymatic oxidation of the hydroxy-methyl group to the carboxyl group leads to the formation of its main metabolite—cetirizine (CZ). CZ is the active substance of antiallergic drugs. Because it does not cross the BBB (blood–brain barrier) easily, it is less likely to cause drowsiness or affect memory and impair cognitive function. Therefore, in criminal studies, it is often important what medication had been taken by a person involved, e.g., in a car accident, HZ or CZ. The analysis of both antihistamine drugs is challenging, as usually very low concentrations of the compound of interest need to be determined. Thus, an ultra-sensitive UHPLC–QqQ-MS/MS method was developed for simultaneous determination of HZ and CZ in biological fluid samples. The lower limit of quantification (LOQ) for HZ and CZ was calculated as 0.345 and 0.3696 ng/mL, respectively. Together with a reduced sample volume to 200 μL, it makes the developed method suitable for a sensitive multidrug forensic toxicological analysis. Samples were extracted with simple and fast liquid–liquid extraction (ethyl acetate, pH 9). The present method for the determination of HZ and CZ in human blood proved to be simple, fast, selective, and sensitive. The quantification by LC–MS/MS was successfully applied to the samples coming from 28 authentic biological fluids (blood, urine, vitreous humor, bile and stomach content), both antemortem and postmortem. The performed studies confirm that the developed method is characterized by a high extraction efficiency. Its accuracy, reproducibility, simplicity, and selectivity suggest its application in clinical, toxicological, and forensic laboratories.

## Introduction

**Fig. 1:**
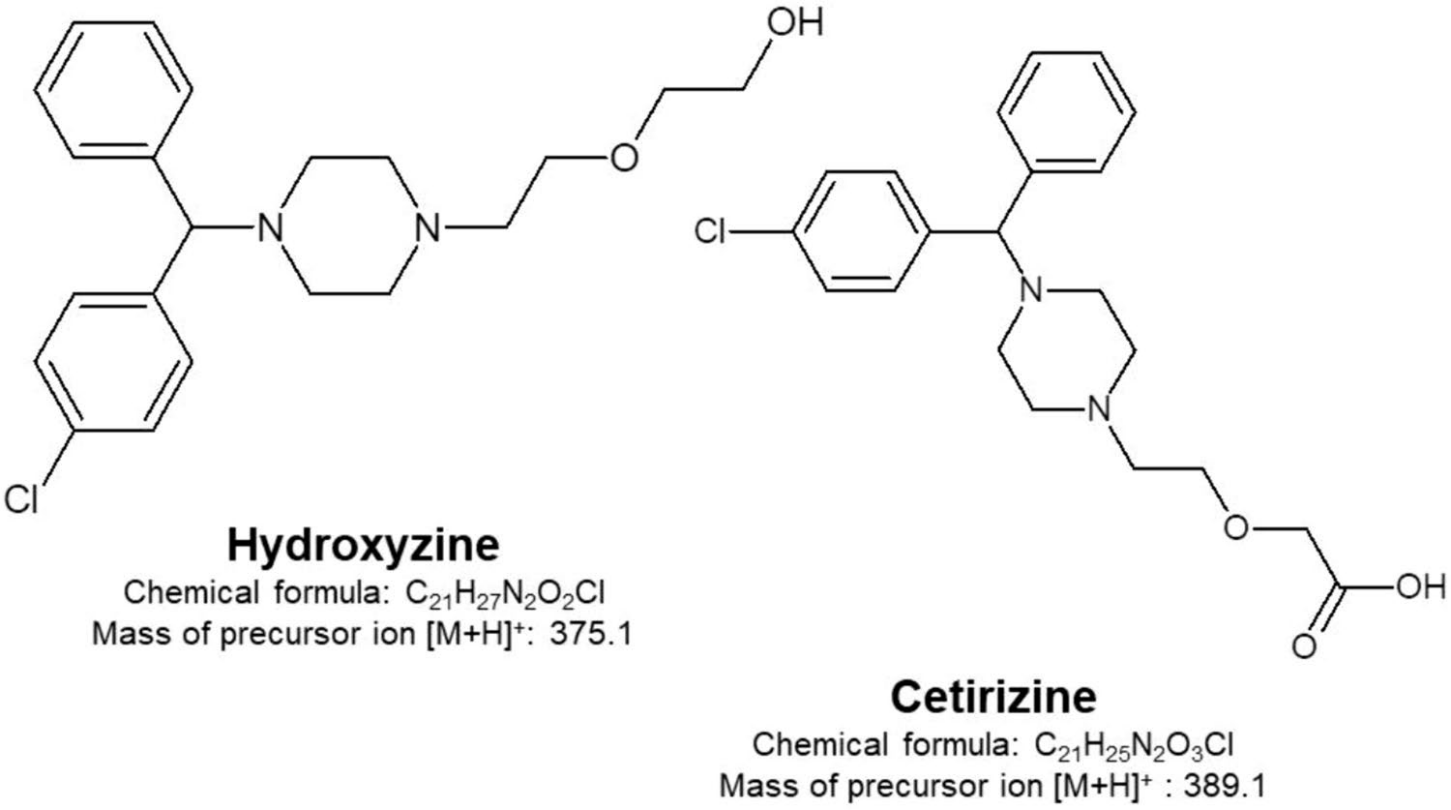
Structures of hydroxyzine (HZ) and cetirizine (CZ) with chemical formula and mass of precursor ion (*m/z*)

Hydroxyzine (HZ) is a multifunctional derivative of piperazine, a basic, highly lipophilic compound with a volume of distribution (Vd) of 13–31 L/kg that undergoes a significant biotransformation and tissue distribution and, thus, easily penetrates the blood–brain barrier (BBB) (Criado et al. 2010). It is commercially available as a hydrochloride salt, usually under the trade names: Atarax®, or Vistaril® (Nojavan and Fakhari 2011). It may be administered in the form of tablets (10, 25, 50 mg), syrups (10 mg/5 mL), oral solutions (10 mg/ 5 mL), injectable forms (25, 50 mg/mL), and as a pamoate capsule (25, 50, 100 mg). Single doses of 25–100 mg and daily doses up to 400 mg may be administered (Baselt 2017). As a first-generation antihistamine drug, HZ (Fig. 1) possesses sedative properties that get enhanced when it is simultaneously administered with other central nervous system (CNS) depressants, such as alcohol, hypnotics, narcotics etc. Thus, it may cause drowsiness, fatigue, dizziness, decreased attention and vigilance, impaired coordination, and slowed reaction time, which may affect the ability to drive and operate machines, resulting in the risk of causing accident (de Jager et al. 2002; Jauregui et al. 2006; Montoro et al. 2006; Simons 2004). It is estimated that drivers who take first-generation antihistamines are six times more likely to have a car accident; for comparison, the use of cell phones in motor vehicles is four times more likely to result in a collision within a short period of time during a telephone conversation (Hindmarch and Shamsi 2001).

In some cases, HZ has been also used to relieve symptoms of opioid (Song et al. 2005) and benzodiazepine (Péhourcq 2004) abuse and alcohol withdrawal syndrome (Tan et al. 2006). HZ is characterized as a strong reversible agonist of the H1 receptor, moderately strong 5-HT2A antagonist, and weak dopamine D2 and α1-adrenergic receptor antagonist. Administered orally, HZ is rapidly absorbed from the gastrointestinal tract and works after 15–30 min with the strongest action after 1 h.

Age, sex, and genetic polymorphisms are important factors leading to a variability in drug metabolism and disposition (Trenaman et al. 2021). HZ undergoes rapid biotransformation in the liver by alcohol dehydrogenase (ADH) and the isoenzymes CYP2D6, CYP3A4 are not substrates of p-glycoproteins (and this distinction may account for their predilection to cause sedation) (Criado et al 2010; Hamelin et al. 1998). Only 0.8% is excreted unchanged by the kidneys. The CYP system can be altered in special metabolic conditions, such as infancy, advanced age, hepatic diseases or by a direct action of other drugs which accelerate or delay the action of these enzymes in the metabolism of H1 antihistamines (Criado et al 2010).

The biological HZ’s half-life in adults is approximately 13–27 h (Baselt 2017). In the case of children in the age group of 6.1 ± 4.6 years, 22.0 ± 12.0 kg b.w., hydroxyzine administered orally in a single dose of 0.7 mg/kg b.w. increases plasma clearance by about 2.5 times compared to adults. In this age group, the half-life of HZ is shorter than in adults due to a faster metabolism, and is about 4–11 h. A mean t_1/2_ of 29.3 h was reported after the administration of 0.7 mg/ kg hydroxyzine syrup to nine healthy, fasting adults of mean age 69.5 years; in patients with hepatic failure, it is extended to 37 h (Trenaman et al. 2021). It has been established that in humans, the total level of CYP enzymes in the liver decreases from about the age of 40. In elderly people, the severity of adverse effects may be increased due to the reduced clearance of drugs whose metabolism is based on CYP3A4 (Trenaman et al. 2021). The hepatic CYP metabolism varied between men and women (Trenaman et al. 2021). In humans, CYP3A4 had a higher level of protein expression in the female liver (Parkinson et al. 2004). An in vitro study on the samples of 43 healthy livers in subjects between the ages of 27 and 83 showed a 24% increase in CYP3A4 activity identified by erythromycin N-demethylation in females (Hunt and Westerkam 1992). Genetic variation in the CYP2D6 gene has been well characterized and 120 CYP2D6 variants (alleles) that have altered levels of CYP2D6 enzyme activity were identified. Phenotypically, individuals with two normal CYP2D6 alleles are extensive metabolizers (EMs), those with one normal and one poor metabolism allele are intermediate metabolizers (IMs) and those with two reduced metabolism alleles are poor metabolizers (PMs). For CYP2D6, there is a fourth phenotype described, from the group of the ultra‐rapid metabolizers (UMs) which have at least one active CYP2D6 gene duplication. In individuals with UM, the original compound may be rapidly and completely converted to a metabolite, which may lead to a higher than expected serum level, increasing the risk of overdose symptoms even at labeled doses (Trenaman et al. 2021). Interestingly, the PM variants are common in East Asian populations and exist across the world (Trenaman et al. 2021). Some antiallergic drugs such as diphenhydramine (a first-generation antihistamine) cause Vd to be 1.7‐fold higher in Orientals compared to Caucasians. Also, unbound diphenhydramine in plasma was higher in Orientals (24%) than Caucasians (14.8%). This provides a potential explanation for the increased Vd in Oriental subjects (Olafuyi et al. 2021).

From a chemical point of view, HZ is an alcohol, i.e., almost half of its metabolism involves enzymatic oxidation of the alcohol group –CH2OH at the end of the protruding side chain of the molecule to the carboxyl group –COOH, which gives the main metabolite: cetirizine (CZ) (see Fig. 1). CZ is a racemic R and S enantiomer mixture. Only one of its enantiomers, i.e., levocetirizine, has strong pharmacological activity toward the H1 receptor and is classified as a second-generation antihistamine drug. CZ is a carboxylic acid metabolite of hydroxyzine. It does not undergo liver metabolization, and therefore does not interact with other drug substances via cytochrome P450 but is a substrates of p-glycoproteins (Bartra et al. 2006). Because it does not cross the BBB easily, it is less likely to cause drowsiness affect memory and impair cognitive function (Criado et al. 2010; Dharuman et al. 2011; Rachelefsky 1998). CZ reaches its maximum concentration in the blood 3 h after oral administration (Tillement 1995). Similarly to hydroxyzine, the age of the patients will play a significant role in the metabolic transformation of CZ. The biological half-life in adults is 8–11 h, while it is reduced to 5–7 h in children (Simons et al. 1989), the biological half-life is prolonged by 50% in older adults and in patients with chronic liver disease as compared with normal healthy adults (Trenaman et al. 2021). It is excreted in the urine in 60–80% unchanged, and about 10% in the feces, 93% bound to serum proteins, Vd totals 0.5–0.8 L/kg (Del Cuvillo et al. 2006; Trenaman et al. 2021).

The wide use of HZ and CZ in medical practice is often associated with its high availability, which in turn may result in frequent poisoning. In many cases of fatal poisoning, HZ or CZ are just one of the xenobiotics present in the autopsy material at concentration levels from therapeutic to lethal. Due to the relatively frequent use of HZ and CZ for suicidal purposes, high concentrations of other xenobiotics can also be detected in the tested material, including sedatives, antidepressants, narcotics, and ethyl alcohol.

The therapeutic range for CZ is 0.11–0.36 µg/mL in plasma and the toxic concentrations are higher than 2.4 µg/mL (Baselt 2017), while a HZ-related fatal case reported a lethal concentration of 39 µg/mL (Ma et al. 2007), although several times lower HZ concentrations were observed, i.e., ranging from 0.7 to 4.2 µg/ml in people who took this drug for suicide purposes (Baselt 2017).

Biochemical and biological processes that occur after death can significantly affect the results of tests in autopsy material. However, the results of postmortem toxicological assays do not always fully reflect the distribution of xenobiotics in the body at the time of death. The assessment of the distribution of drugs and their metabolites in fatal poisonings is a leading issue in the field of forensic medical judgments aimed at formulating an opinion on the cause of death (Abdelaal et al. 2023). Postmortem redistribution (PMR) should be mentioned here, which describes anatomical and physiological changes that can falsely alter concentrations after death (Brockbals et al. 2018). The degree of redistribution depends on the physicochemical properties of the xenobiotic, the time from death to the moment of sample collection, the time that has elapsed since death, the cause of death and the fate of the organism immediately before it (e.g., stomach contents), and atmospheric factors (temperature and humidity) (Han et al. 2012; Shintani-Ishida et al. 2018). Typical reservoirs in which drugs subject to PMR can accumulate during life are the liver, lungs, and heart (Abdelaal et al. 2019). Postmortem redistribution also occurs from blood in the femoral veins (a material that toxicologists generally consider to be the most resistant to any postmortem changes) (Øiestad et al. 2018).

The concentration of many drugs determined postmortem in blood from the heart is usually higher from the concentrations determined in peripheral blood (e.g., from the femoral vein), which may be a consequence of PMR. Depending on the type of xenobiotic, its concentration in the blood may increase or decrease after death. An increase in the concentration is observed for drugs that easily bind with the tissues surrounding the blood vessels during life, and are quickly released from them into the blood after death. In the case of hydroxyzine, it may diffuse along the concentration gradient from the gastrointestinal lumen to the nearest tissues and organs with good blood supply (including peripheral blood, vitreous body, urine, liver, lungs, kidneys, brain, heart), due to properties such as easy penetration through biological membranes, low affinity to proteins, good solubility in water (similar to barbiturates, tricyclic antidepressants). In addition, hydroxyzine—due to its physicochemical properties and that its Vd is higher than greater than 3 L/kg—may be more prone to PMR (Yarema and Becker 2005). Estimating whether the concentration of a drug in peripheral blood found in postmortem examinations was higher or lower than during life solely based on the proportion of the concentration of the xenobiotic between the cardiac blood and peripheral blood ratio (C/P) is not evidential, although it is a commonly used marker for the prediction of PMR of drugs in forensic autopsies (Emaus et al. 2023). Large differences in C/P values have been found, and it has not yet been determined which C/P value indicates significant PMR and which one indicates minimal PMR. Hydroxyzine does not appear to exhibit significant postmortem redistribution. A ratio of 1.0 has been reported in two fatalities, as well as in four other cases, but in the McIntyre study, the C/P for hydroxyzine was in the range of 0.57–1.27 (McIntyre et al. 2013) and in the Dalpe-Scott study, it was in the range of 0.50–1.5 (Dalpe-Scott et al. 1995). Based upon the C/P ratio model, the possibility of some degree of PMR occurring in the peripheral blood cannot be discounted (McIntyre 2016).

Another more reliable way to assess PMR is the proportion between the concentration of hydroxyzine in the liver and its concentration in peripheral blood (Liver to peripheral blood ratio L/P), the L/P ratio has been proposed as a marker for PMR, with ratios < 5 indicating little to no propensity for redistribution and ratios > 20–30 indicative of PMR (Kennedy 2015; Özşeker 2015). In McIntyre’s study, in ten cases, the postmortem L/P was in the range of 7.4–25, which may indicate that hydroxyzine is prone to a moderate degree of postmortem redistribution (McIntyre et al. 2013). This ratio is analogous to those previously reported in the cases of hydroxyzine fatal overdose: in the studies of Johnson (Johnson 1982) who showed a ratio of 10.6 or in the tests of Spiehler and Fukumoto (Spiehler and Fukumoto 1984) who established a ratio of 14.5. According to other literature data, for HZ, it has been demonstrated that PMR does not occur (Chambers et al. 2007; Leikin, Watson 2003).

A number of methods are currently applied for the analysis of HZ and CZ in various biological samples, including blood, plasma, urine, stomach content, bile, vitreous humor or different tissues, e.g., muscles. Some determinations are performed with help of spectrophotometric (Rajendraprasad et al. 2011) or voltammetric (Beltagi et al. 2008) techniques; however, these are often not enough sensitive nor selective. High-resolution techniques that can be suitable for the quantitative determination of drugs in biological samples are currently on demand. That is why forensic medicine aims at the utilization of chromatographic methods that bring reliable results for the identification and quantification of drugs including those impairing psychomotor performance. Chromatographic techniques that include liquid chromatography (LC)-based methods with mass spectrometric (de Jager et al. 2002; Eriksen et al. 2002; Kim et al. 2005; Péhourcq 2004; Song et al. 2005), fluorescent (Hammad et al. 2007), or UV–Vis detection (Hammad et al. 2007), gas chromatography (GC) with electron capture (Hartvig and Handl 1975), GCMS (Fouda et al. 1979), but also capillary zone electrophoresis (Fortes et al. 2013) have been involved in various studies described in the scientific literature. The aforementioned methods are precise but they require a time-consuming sample preparation protocol. In forensic toxicology, it is necessary to develop a simple and quick analytical procedure that guarantees reliable results. These can include electrospray ionization (ESI), or atmospheric pressure chemical ionization (APCI) which—together with a triple quadropole MS/MS-based detection—offer high sensitivity and selectivity of quantitative determination.

Unfortunately, many of previously published protocols involved the use of very large volumes of biological material (Fortes et al. 2013; Kim et al. 2005; Péhourcq 2004), which in terms of forensic toxicology and postmortem examinations may pose a significant problem, especially when only a small amount of blood was collected for testing, e.g., in accidents with exsanguination (Szpot et al. 2023) or in the cases related to neonates (Szpot et al. 2022).

Both postmortem toxicological and medical-forensic examinations are very important in the case of taking various types of chemical substances. Recently, it has become possible to lower the limit of detection (LOD) in biological specimens. However, the interpretation of the results and the established standards for method validation require much more attention to toxicological analysis; hence, many of the case reports published in recent years contain detailed data on analytical problems accompanying postmortem examination (Wachełko et al. 2023; Zawadzki et al. 2021a, 2021b, 2021c).

This paper aims to develop an ultra-sensitive UHPLC–QqQ-MS/MS method for simultaneous determination of HZ and CZ in biological fluid samples. The developed method was fully validated and used for the determination of HZ and CZ in the authentic blood samples collected postmortem in forensic laboratories.

## Materials and methods

### Chemicals and reagents

The following chemicals and reagents were used in the chromatographic analysis: acetonitrile, methanol, water (Chromasolv® LC–MS, Honeywell Riedel-de Haën™, Seelze, Germany), formic acid (Honeywell Riedel-de Haën™, Seelze, Germany), CZ, HZ (Sigma-Aldrich, St. Louis, USA), CZ-d8 (Cat no. HY-17042S1-1 mg), HZ-d8 (Cat no 014674-1 mg) (Toronto Research Chemicals INC, North York, Canada), ethyl acetate (Chromasolv® LC–MS, Honeywell Riedel-de Haën™, Seelze, Germany), ammonium carbonate (Sigma-Aldrich, St. Louis, USA). Standard solutions of CZ, HZ and internal standards (ISTD) CZ-d8, HZ-d8 were prepared in methanol, and further diluted to obtain the working solutions of different concentrations. All solutions were stored at − 20 °C.

### Biological material

Drug-free blank blood samples used for the development and validation of the method were obtained from a regional blood donation center (Wrocław, Poland). Drug-free blank urine samples used for the development and validation of the method were obtained from a healthy volunteer. Blank samples were screened before spiking to ensure that they were free from CZ and HZ. Forensic biological fluids (blood, urine, vitreous humor bile and stomach content) were sent to our laboratory for toxicological analyses.

### Sample preparation

The sample procedure was carried out according to the previously described procedure by Zawadzki et al. (2021a) with minor modifications. Prior to the chromatographic analysis, the sample preparation procedures were implemented to remove the interferences from the matrix. In this case, the liquid–liquid extraction-based techniques were used. For the purpose of the study, the aliquots of 200 μl of biological material (blood, urine, vitreous humor) were transferred to 12 ml volume tubes with 20 μl of internal standards’ solution (CZ-d8 and HZ-d8 at the concentration of 1 μg/ml). Then 200 μl of buffer (0.5 M ammonium carbonate, pH 9) was added and the liquid–liquid extraction of the described mixture with 2 ml ethyl acetate was carried out for the succeeding 10 min. The samples were later centrifuged at 2500 × g at 4 °C for 10 min and the organic phase was transferred to 2 ml Eppendorf tubes. The content was then evaporated to dryness under a stream of nitrogen (at 45 °C) and the dried residue was re-dissolved in 50 μl of methanol. The obtained solution was used directly for the analysis. Because the concentrations of hydroxyzine and cetirizine in some biological fluids were markedly above ULOQ (upper limit of quantification), the assay was repeated and samples were diluted tenfold and 100-fold with water (LC–MS grade).

### Instrumentation

#### HPLC–APCI-QqQ-MS/MS analysis (Method 1)

Chromatographic analysis was performed using a high-performance liquid chromatograph (HPLC 1260, Agilent Technologies, Waldbronn, Germany). The detection of the investigated compounds was achieved using a triple quadrupole mass spectrometer (QqQ 6460, Agilent Technologies, La Jolla, USA) equipped with APCI. Prior to the analysis, the optimization of the chromatographic conditions was performed. First the mixture of standards was analyzed in acetonitrile and methanol-based gradients—with different content of formic acid, namely 0.1, 0.2, and 0.3%. Also, the selection of the chromatographic columns that differed with length (50, 100 and 150 mm length) was performed on the analyzed samples. After all, the chromatographic separation was performed employing a Poroshell 120 EC-C18 column 3.0 × 50 mm; 2.7 μm (Agilent Technologies, La Jolla, USA) with a thermostat at 35 °C. A mixture of 0.1% formic acid in water (A) and acetonitrile (B) was used as a mobile phase. The injection volume was 10 μl. A flow rate of 0.5 ml/min was used. Gradient elution was as follows—0 min: 5% (B), 4.5 min: 60% (B), 5 min: 95% (B), 6.5 min: 95% (B). The column was re-equilibrated for 2.5 min. after the gradient separation. The quantitative analysis was carried out in a dynamic Multiple Reaction Monitoring (dynamic-MRM) mode (Table). The following MS parameters were fixed—gas temperature: 325 °C; gas flow: 4 L/min.; vaporizer: 350 °C; nebulizer: 20 psi; capillary voltage: 4500 V; corona current: 4 µA; cell accelerator voltage: 4 V. Recording and handling of data in the Method 1 were performed using an Agilent Mass Hunter Optimizer software (Agilent Technologies, La Jolla, USA). A summary of precursor and product ions, collision energies, fragmentor, and retention time for each compound is presented in Table 1.

**Table 1:** Multiple Reaction Monitoring (MRM) conditions used in the present HPLC–APCI-MS/MS analysis of CZ, HZ, and CZ-D*8*, HZ-D*8* used as internal standard (Method 1)

Compound	Precursor ion [*m/z*]	Product ion [*m/z*]	Collision energy [V]	Fragmentor [V]	Retention time [min]
CZ	389.2	201.1	68	60	5.58
		166.1	12		
CZ-D8	397.2	166.1	52	104	5.57
HZ	375.2	201.1	20	124	5.48
		166.1	48		
HZ-D8	383.2	166.1	68	92	5.47

#### UHPLC–ESI–QqQ-MS/MS analysis (Method 2)

Chromatographic analysis was performed using an ultra-high-performance liquid chromatograph (UHPLC Nexera X2, Kyoto, Japan). Detection of the investigated compounds was achieved using a triple quadrupole mass spectrometer (QqQ 8050 Shimadzu, Kyoto, Japan) equipped with ESI. Separation was done employing an Acqutity UPLC® BEH C18 column 2.1 × 50 mm; 1.7 µm (Waters, Wexford, Ireland) with a thermostat at 40 °C. A mixture of 0.1% formic acid in water (A) and 0.1% formic acid in acetonitrile (B) was used as a mobile phase. The injection volume was 1 μl. A flow rate of 0.5 ml/min was used. Gradient elution was as follows—0 min: 5% (B), 4.5 min: 60% (B), 5 min: 95% (B), 6.5 min: 95% (B). The column was re-equilibrated for 2.5 min, after the gradient separation—5% (B). Quantitative analysis was carried out in a MRM mode (Table). The following MS parameters were fixed—interface temperature: 300 °C; nebulizing gas flow: 3 l/min.; DL temperature: 250 °C; heating gas flow: 10 l/min; heating block temperature: 350 °C; drying gas flow 10 l/min; dwell time: 9 s. The acquisition of spectra and the analysis of data was achieved using the Lab Solutions program (Shimadzu, Kyoto, Japan). A summary of precursor and product ions, collision energies, Q1-Q3 pre-bias voltages, and retention time for each compound is presented in Table 2.

**Table 2:** Recovery, matrix effects, accuracy, and precision (Method 1)

Concentration [ng/ml]	Recovery [%]	Matrix effects [%]	RSD [%]	MRE [%]
Cetirizine 10 100 500				
	100.5	0.5	5.3	− 1.7
	93.9	− 6.1	1.4	− 5.0
	99.3	− 0.7	10.5	15.0
Hydroxyzine 10 100 500				
	90.8	− 9.2	2.1	11.3
	98.7	− 1.3	6.7	− 1.1
	94.6	− 5.4	7.6	− 1.1

### Validation

Sample preparation, validation of the method and analytical conditions were identical to those described previously by (Zawadzki et al. 2021a, b, c). Validation process included examination of selectivity, linearity, precision and accuracy, carryover, lower and upper limits of quantification, recovery, matrix effect, and process efficiency. The selectivity of the method was evaluated by analyzing blank human blood and blank blood spiked with CZ, HZ, and internal standards. Calibration curves were prepared by spiking working solutions with appropriate volume ratio into the treated blood, to yield the concentrations of 5, 10, 50, 100, 200, 500, and 1000 ng/ml. The coefficient of determination (*R*^2^) was determined. According to the acceptance criteria used, the coefficient of determination should meet the condition: *R*^2^ ≥ 0.995. To achieve precision and accuracy, four repeats of CZ and HZ spiked blank human blood at 10 (low QC), 100 (medium QC), and 500 (high QC) ng/ml were analyzed with calibration samples in one batch. Precision was defined as relative standard deviation (RSD%), while accuracy was expressed as mean relative error (RE%). The lower limit of quantification (LLOQ) was defined as the concentration at which the relative standard deviation (RSD%) and relative error (RE%) do not exceed 20% and 15%, respectively, of the area ratio observed for the LLOQ samples (Peters et al 2009). The LOD was considered to be the lowest concentration of the sample for which the signal to noise ratio met the condition at least: S/N ≥ 3. The limit of quantification (LOQ) was calculated as a multiple of LOD, i.e., LOQ = 3xLOD. The recovery and matrix effect were evaluated at each of the three different concentrations: 10, 100, and 500 ng/ml. The recovery (%, *n* = 5) was calculated at time zero and after 24 h. The recovery of CZ and HZ was determined using the ratio of analytical signal from four repeats of each CZ and HZ extract concentration compared to the signal from non-extracted methanol standards of equal concentrations. The matrix effect (% bias, *n* = 5) was determined by comparing the response of analyte spiked after the extraction of blank matrix with the response of analyte in neat solution and calculated using an equation described by (Chambers et al. 2007):

$$\% {\text{ Matrix effects }} = \left( {\frac{Response extracted sample}{{Response standard}} - 1} \right){*}100$$

## Results and discussion

**Fig. 2:**
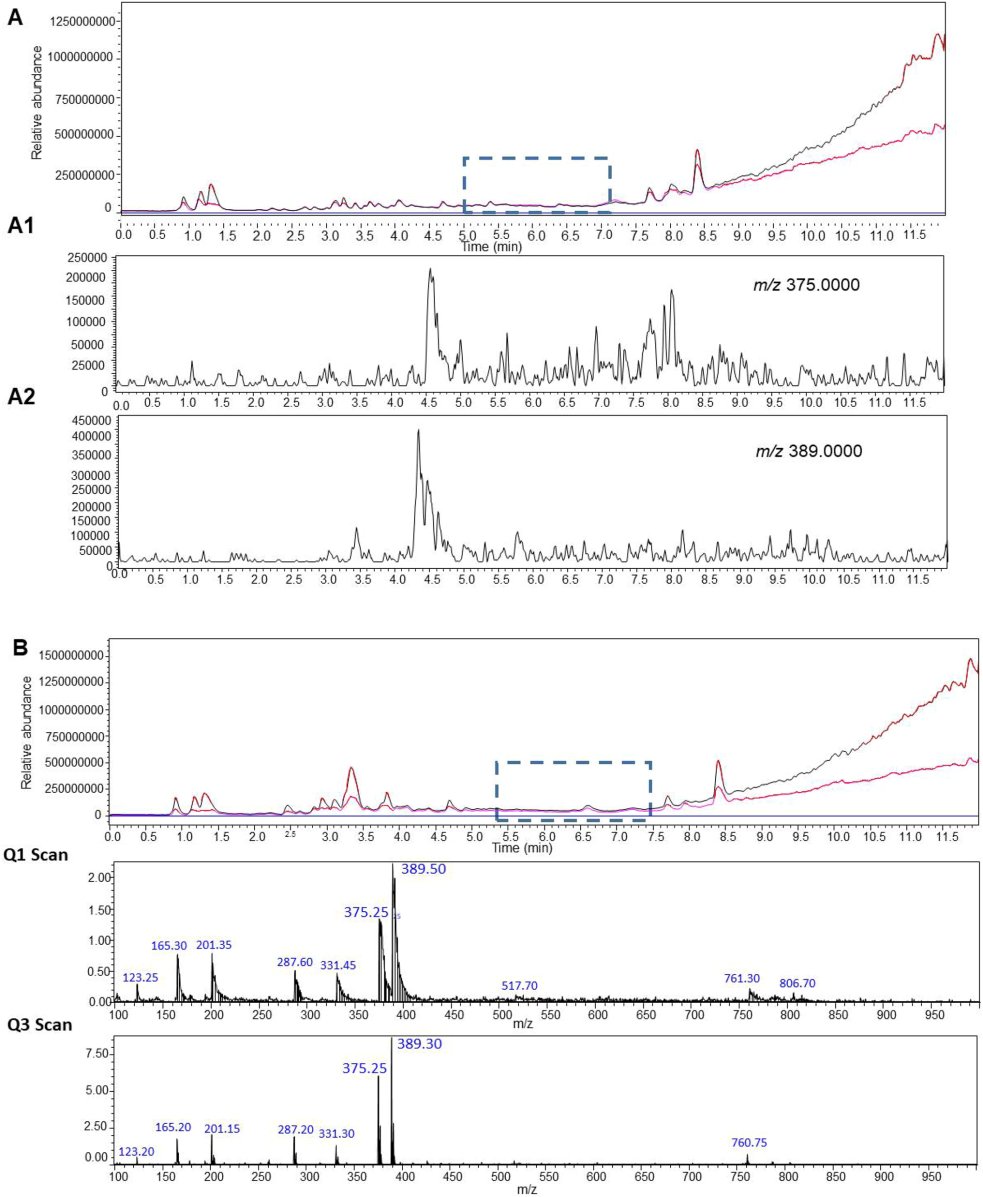

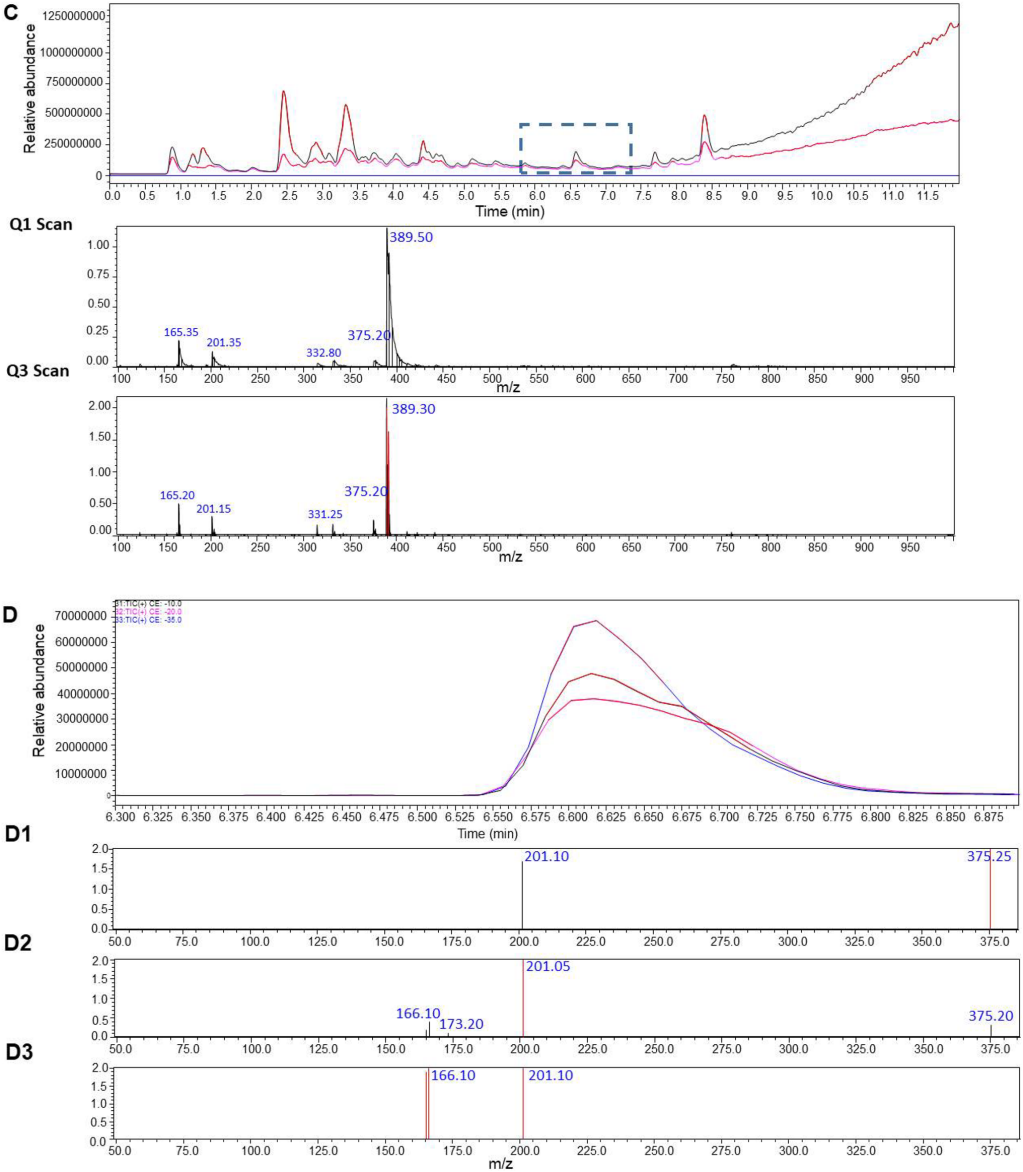

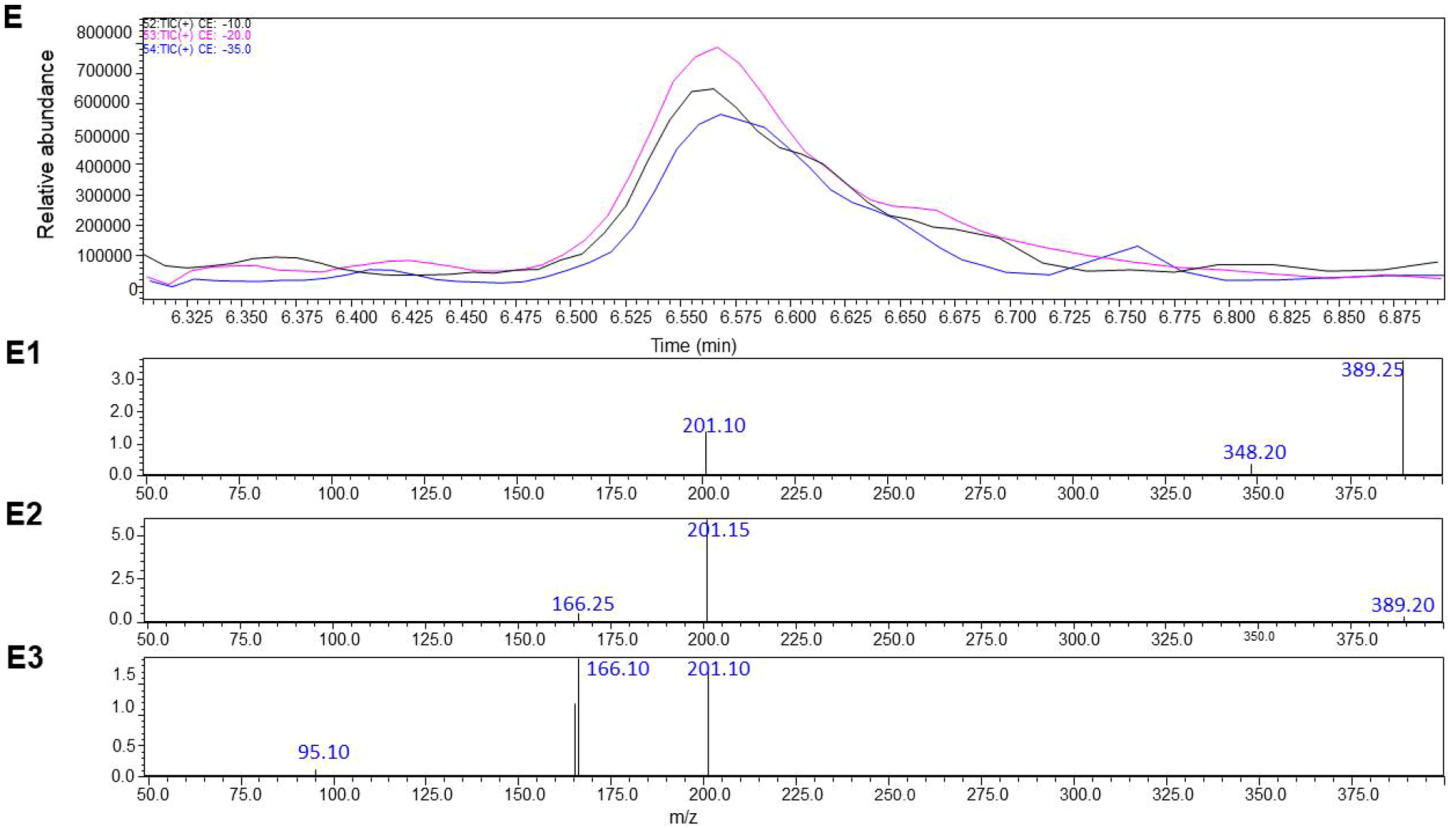
The targeted LC–MS/MS chromatograms obtained for blank matrix (**A**) with isolation m/z precursor ion for HZ (A1) and CZ (A2), scan of an authentic blood sample (**B**), scan of an authentic urine sample (**C**), product ion spectra of HZ in authentic human blood; collision energy: a –10, b –20, and c –35 V (**D**, D1–D3), product ion spectra of CZ in authentic human blood; collision energy: a –10, b –20, and c –35 V (**E**, E1–E3)

A simple liquid–liquid extraction was successfully applied to extract the HZ and CZ and the IS from postmortem samples. No interfering ion current signals were observed at the retention time of this compounds (Fig. 2A). The optimized conditions of the sample preparation that were elaborated in this project provided clear mass spectra with low matrix effect in the tested real forensic samples. In Fig. 2, a sample chromatogram of urine and blood with marked peaks of HZ (eluted at 6.67 min) and CZ (eluted at 6.5 min) is presented (Fig. 2 B and C). The elaborated MRM parameters, fragmentor voltage and collision energies that are suitable for the determination of HZ and CZ (and respective standards) in biological samples were optimized for both proposed methods employing the usage of mass detectors with different ionization sources. The optimal parameters for both instrument types are presented in detail. Table 1 displays the validation parameters of the method employed. All presented values fall within the acceptable range for toxicological analysis of biological materials, in accordance with the recommendations of the German Society of Toxicological and Forensic Chemistry (GTFCh) (Peters et al. 2009). As a result, the method has been successfully implemented for routine toxicological analysis in our laboratory.

### Method 1

Method 1 was performed using an instrument composed of an HPLC chromatograph coupled with an APCI-QqQ-MS/MS.

**Fig. 3:**
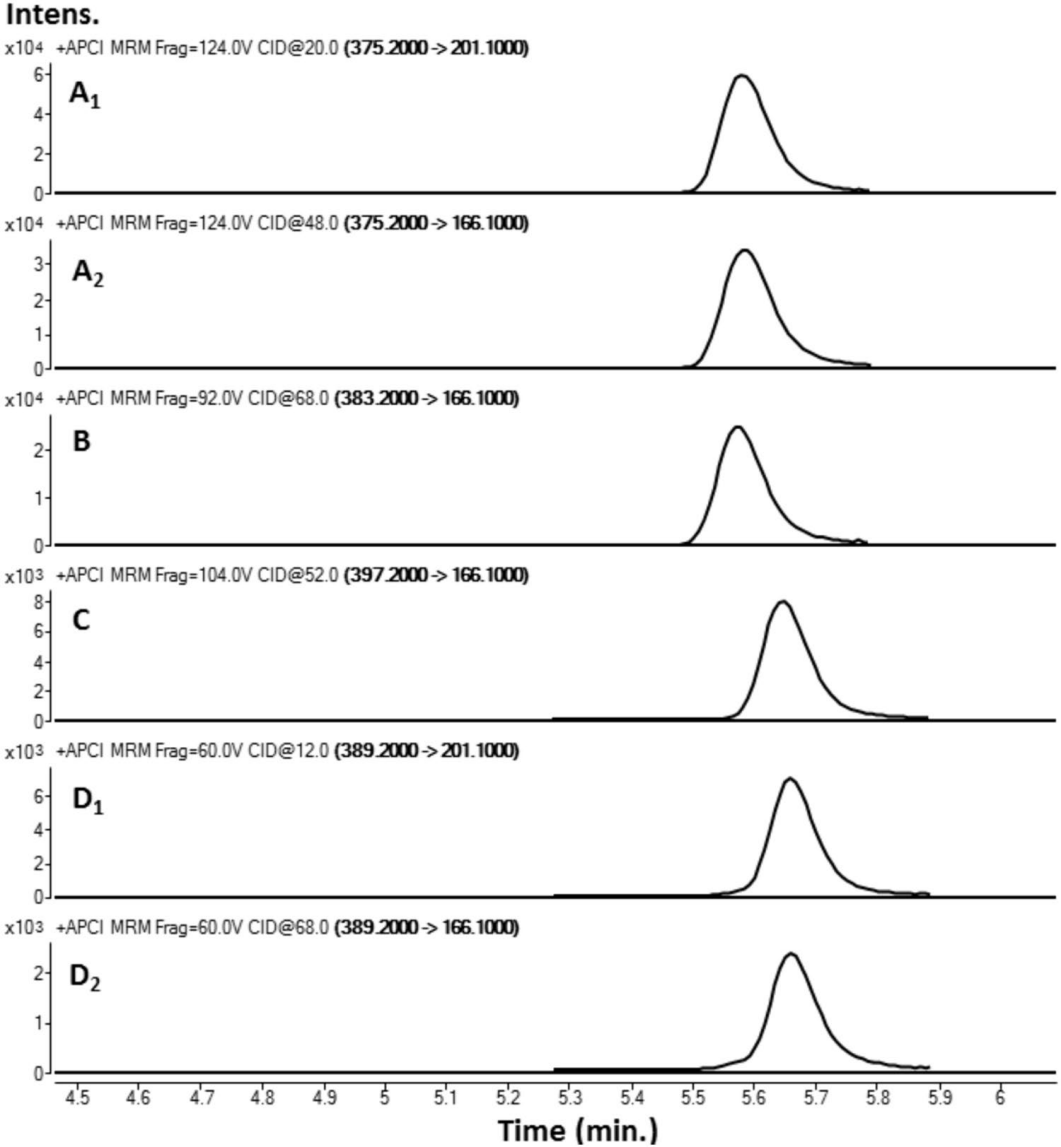
Dynamic MRM chromatograms (Method 1) of blood sample spiked (at concentration 100 ng/ml) with HZ (A1-2), HZ-d8 (**B**), CZ-d8 (**C**), and CZ (D1-2)

The operating platform was checked for its selectivity, linearity, LOQ, precision, accuracy, recovery, and stability. As a result of the selectivity assay, no interfering endogenous substances were observed at the retention times of the analytes and ISTD (see Fig. 3). Concerning the linearity of the results, the quadratic and linear regressions of the peak area ratios versus concentrations were fitted over the concentration range 5–1000 ng/ml for CZ and HZ in human blood. The following calibration curve equations were obtained: y = 2.298011 x—0.023062 for HZ and y = − 0.035042 x^2^ + 0.843162 x—0.028029 for CZ and the coefficient of determination *R*^2^ was calculated as *R*^2^ ˃ 0.9998 for both compounds, respectively, showing high repeatability of the composed method and precision of the taken measurements. It is important to underline that the calibration equation calculated for CZ is not linear; however, the results from the analyses of the tested samples were collected from the area of the lowest steepness of the curve. Concerning the LLOQ for both drugs in human blood samples, it was calculated as 0.3696 ng/ mL for CZ and 0.345 ng/ mL for HZ. The LOD for these drugs was equal to 0.1232 and 0.1150 ng/mL, respectively. Precision, accuracy, recovery, and stability results of this method are shown in Table 2. As presented below, the recovery rate of HZ and CZ exceeded 90%, which makes this method a reliable one.

As it is shown in Table 1 both drugs delivered similar fragments from their molecular ions, namely 166.1 and 201.1, which shows their similar chemical structure and the scaffold they are originating from. The results prove that even if the compounds are of similar chemical nature (similar retention time in the applied chromatographic column), HZ is harder ionizable from CZ as the optimal fragmentation energy was set as 124 V in comparison to CZ, which was easily fragmented and its fragmentation value was determined as 60 V in the used instrument equipped in the APCI source.

Among the elaborated methodological conditions, the optimal collision energy values that were used to observe a clear fragmentation of the molecules of interest were ranging between 12 and 68 V. The fragmentation of CZ to the 166.1 m*/z* ion was easier and demanded a significantly lower collision energy, namely 12 V in comparison with the 201.1 ion that was obtained at 68 V. The results for CZ were opposite from those recorded for HZ, where the fragmentation of the molecular ion to the *m/*z of 201.1 was easier and appeared at its highest efficiency at 20 V, in comparison to the voltage of 48 V that was necessary to receive the 166.1 signal. In both cases, the deuteration of a molecule leads to the necessity for the elevation of the fragmentation energy value to obtain similar fragments as in the un-deuterated molecule.

### Method 2

**Fig. 4:**
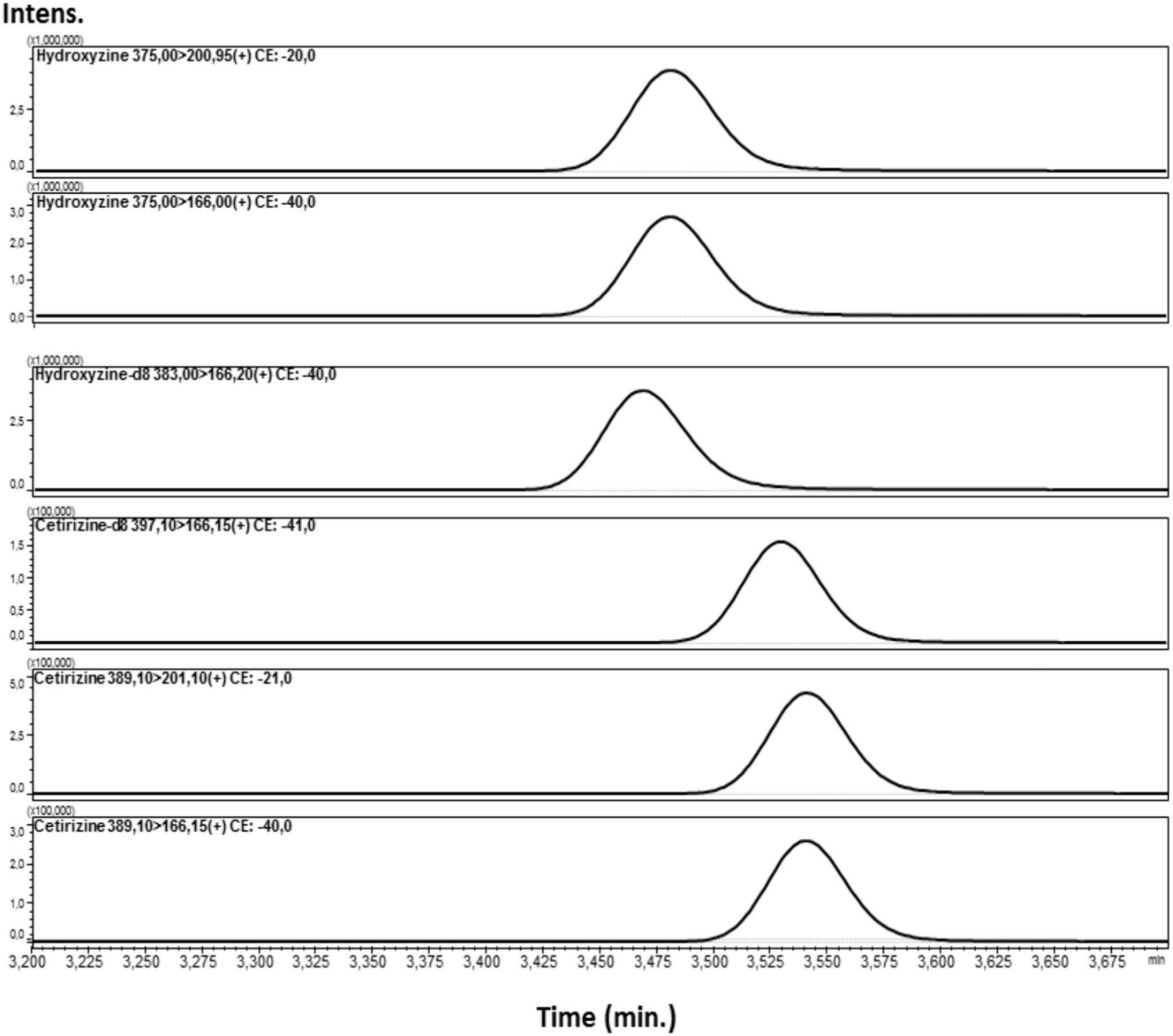
MRM chromatograms (Method 2) of blood sample spiked (at concentration 100 ng/ml) with HZ, HZ-d8, CZ-d8, and CZ

Different analytical parameters needed to be set when using another ionization source. The optimized conditions suitable for the determination of the two drugs, HZ and CZ using the ESI ionization source, were elaborated in a similar manner as for the Method 1. First, the selectivity studies performed on the tested solutions did not reveal the presence of any other interfering endogenous substances that could be washed out from the chromatographic column at a similar time as the analytes and ISTD (see Fig. 4). In the following studies, the linearity of the tested concentrations was studied. As a result, the linear regressions of the peak area ratios versus concentrations were fitted over the concentration range 5–1000 ng/ml for CZ and 5–500 ng/ml for HZ in human blood samples. The obtained calibration curve equations were: y = 0.589749x + 0 for HZ and y = 1.78190x + 0 for CZ. As a result, the calculated R^2^ coefficients exceeded 0.999 for both compounds assuring a high precision and low standard deviation values of the obtained data. The calculated LLOQ for CZ and HZ in human blood was 1 ng/mL. This result confirms high sensitivity of the elaborated protocol which is particularly necessary when analyzing biological samples. The LOQ for the ESI source is the same as for the APCI ionization method. The detailed parameters of the optimization, like precision, accuracy, recovery, and stability results of this method, are presented in Table 3. As previously, the applied conditions show high recovery rates of the drugs of interest.

**Table 3:** Recovery, matrix effects, accuracy, and precision (Method 2)

Concentration [ng/ml]	Recovery [%]	Matrix effects [%]	RSD [%]	MRE [%]
Cetirizine				
10	100.9	0.9	2.5	− 8.1
100	104.3	4.3	4.8	1.6
500	93.7	− 6.3	3.4	− 1.5
Hydroxyzine				
10	108.6	8.6	1.2	− 2.6
100	110.3	10.3	0.5	3.0
500	105.2	5.2	0.4	− 0.5

The analysis of the fragmentation pattern of HZ and CZ brought similar results as in the APCI-based Method 1. The compounds delivered two distinct fragment ions with *m/z* of 166.15 and 201.15. The MRM transitions of parent ions to product ions were measured at different energy settings. As a result, the collision energies of − 21 and − 40 were selected as the most favorable for the formation of the 201.1 and 166.15 fragments from CZ, respectively, whereas the energies of − 20 and − 40 were selected for the daughter ions of HZ, respectively. Close values obtained for both drugs confirm their similar chemical character and an alike behavior in the electrospray (see Table 4).

**Table 4:** Multiple reaction monitoring conditions used in the present UHPLC/ESI–MS/MS analysis of CZ, HZ and CZ-D*8*, HZ-D*8* used as internal standard (Method 2)

Compound	Precursor ion [*m/z*]	Product ion [*m/z*]	Collision energy [V]	Q1 pre-bias [V]	Q3 pre-bias [V]	Retention time [min]
CZ	389.1	201.10	− 21	− 11 − 11	− 20 − 16	3.55
		166.15	− 40			
CZ-d_8_	397.1	201.15	− 21	− 11 − 11	− 20 − 16	3.54
		166.15	− 41			
HZ	375.0	200.95 166.00	− 20 − 40	− 20 − 17	− 20 − 20	3.48
HZ-d_8_	383.0	201.10 166.20	− 21 − 40	− 17 − 18	− 20 − 21	3.49

The developed LC–MS method was optimized and validated for the simultaneous determination of HZ and CZ in whole blood samples. A significant number of HZ- and CZ-related intoxications concerning accidental poisonings of infants (Simons 2004), as well as several fatalities due to HZ overdose, either due to accident (McIntyre et al. 2013) or committing suicide (Ma et al. 2007), have been described in the scientific literature. As a result, the determination of the above antihistamines in postmortem samples during the investigation of relative forensic cases is needed. Two methods that are presented in this paper can be used for the simultaneous identification of HZ and CZ in whole blood samples and could be also applied, after proper validation, to plasma, serum, urine or gastric content samples for forensic purposes during investigation of respective cases (Nojavan et al. 2012).

To the best of the authors’ knowledge in the scientific literature, there are only two works that relate to the determination of CZ and HZ in human blood (Gergov et al 2001; McIntyre et al. 2013). However, for those studies five times more blood was required in comparison with the method presented in our paper. In the work published by Gergov et.al. (Gergov et al 2001), a LLOQ (1 ng / ml for HZ and CZ) was achieved but with using a time-consuming two-step liquid–liquid extraction. Another drawback of this work is that five times larger volume of biological material was required for conducting an analysis than in our described methods.

An additional advantage of the method described in this paper is the use of two MRM transitions to identify CZ and HZ. So far, all authors have used only a single transition (de Jager et al. 2002; Eriksen et al. 2002; Gergov et al 2001; Kang et al. 2010; Ma et al. 2007; Song et al. 2005). In forensic toxicology, the internal standard technique is the most common one to determine the quantity of a compound of interest in the sample. The use of deuterated internal standards provides high recovery of marked analytes, comparable to the results of the authors who have applied levoceterizine-D8 (Kang et al. 2010), indometacin (Kowalski and Plenis 2007), phenylalanine (Ma et al. 2007), nebivolol (Dharuman et al. 2011), oxybutynin (de Jager et al. 2002) or HZ (Eriksen et al. 2002). The application of HZ as an internal standard may not be desired for the sample itself as the individuals from the tested groups may also take HZ on a daily basis (see Table 5).

**Table 5:** Comparison of LC–MS methods for determination of HZ and CZ in biological samples

Biological sample (volume)	Sample preparation	Instruments	Internal standard	Linearity range [ng/mL or ng/g]	LOQ [ng/mL or ng/g]	References
Plasma (200 µl)	Solid-phase extraction with Oasis HLB cartridges	LC–MS/MS	HZ	–	5.0	Eriksen et al. 2002
Plasma (100 µl)	Solid-phase extraction with Oasis HLB cartridges	HILIC–MS/MS	CZ-*d*_*8*_^a^	1.00–1000 ng/ml	1.00	Song et al. 2005
Plasma (1000 µl)	LLE (hexane-isoamylic alcohol)	HPLC–UV	Clothiapine^b^	20–1500 ng/ml	20.0	Péhourcq 2004
Plasma (200 µl)	Protein precipitation with ACN	HPLC–ESI–ion trap mass spectrometry	Tramadol^a^	10–1000 ng/ml	–	Tan et al. 2006
Plasma (100 µl)	Protein precipitation with ACN	LC–MS/MS	Oxybutynin^a^	–	0.5	de Jager et al. 2002
Plasma (200 µl)	Protein precipitation with MeOH	HPLC/MS/MS	Phenylalanine^a^	1.0–400 ng/ml	1.0	Ma et al. 2007
Plasma (150 µl)	LLE (methylene chloride/diethylether)	LC with photodiode array detector	Nebivolol^a^	2 to 450 ng/ml-plasma 1–500 ng/ml –urine	2.0 (plasma) 1.0 (urine)	Dharuman et al. 2011
Plasma (300 µl)	Two step: protein precipitation with ACN and LLE (hexane–dichloromethane)	LC–MS/MS	Levoceterizine-*d*_*8*_^a^	0.5–300 ng/mL	0.5	Kang et al. 2010
Plasma (1000 µl)	LLE (methylene chloride)	LC–MS/MS	Methyl paraben^a^	10–400 ng/ml	–	Kim et al. 2005
Plasma (1000 µl)	Dispersive liquid–liquid microextraction (DLLME)	Capillary electrophoresis(CE)	Risperidone	250–12500 HZ 125–6250—CZ	125.0 for HZ 250.0 for CZ	Fortes et al. 2013
Blood (200 µl)	LLE (pH 9.0; ethyl acetate)	LC–QqQ-MS/MS	CZ-*d*_*8*_ and HZ-*d*_*8*_	Non-linear	5.0	Presented Method 1
Blood (200 µl)	LLE (pH 9.0; ethyl acetate)	LC–QqQ-MS/MS	CZ-*d*_*8*_ and HZ-*d*_*8*_	5–1000 ng/ml- CZ 5–500 ng/ml- HZ	5.0	Presented Method 2

information not provided; ^a^—method which was used for the determination of CZ only, ^b^—method which was used for the determination of HZ only

*ACN* acetonitrile, *HZ *hydroxyzine, *CZ *cetirizine, *LOQ *limit of quantification, *LC* liquid chromatography, *MS* mass spectrometry, *MS/MS* tandem mass spectrometry, *SPE* solid-phase extraction, *LLE* liquid–liquid extraction

The determination of very low levels of drugs in the biological material is a challenge for forensic and clinical toxicologists. While elaborating new analytical methods, next to achieving the lowest possible LLOQ value, it is important to make it simple and fast. Table 5 sets together analytical methods that were elaborated for the determination of HZ and CZ content in biological samples. As presented in Table 5, the majority of scientific papers rely were on liquid chromatography coupled with mass spectrometry.

Concerning the biological material that has to be examined, due to its complexity, a separation of analytes from the endogenous components of the matrix (e.g., proteins, phospholipids, salts, acids or bases) and impurities (like putrefaction products) is necessary to diminish the load on the chromatographic system. The described methods of the samples’ preparation are diverse and depend on the laboratory. As presented in Table 5, one of the most commonly used preparation technique was precipitation (de Jager et al. 2002; Ma et al. 2007; Tan et al. 2006). Eriksen et al. (2002) and Song et al. (2005) used the procedure of solid-phase extraction an Oasis HLB adsorbent.

In the following two publications (de Jager et al. 2002; Kang et al. 2010) the LOQ of 0.5 µg/L was obtained. In these papers, the authors used precipitation technique, whereas the lowest volume suitable for a successful determination of the analytes was 100 µl (de Jager et al. 2002). On the other hand Kang et al. (2010) used 300 µl of a sample in a two-step procedure including extraction, precipitation, and LLE extraction. In their works, Song et al. (2005) and Ma et al. (2007) described a method that is characterized by LOQ = 1.0 µg/L.

In these cases, 200 μL of biological material were used for extraction, similarly to the herein presented method. The important difference between the methods described in these works and the protocol we propose lies in the use of a different method of sample preparation. Both Eriksen et al. (2002) and Song et al. (2005) used SPE and OASIS, respectively, which is a more time-consuming method than the simple liquid–liquid extraction we applied. LLE is characterized by a short time of preparation, high efficiency, and low operational cost.

An additional advantage of the proposed protocol is the possibility of using it for any type of biological material (including blood, serum, plasma, urine, sections of internal organs and others). Kang et al. in their method managed to achieve the lower LOQ of 0.5 µg/L (Kang et al. 2010). The procedure they developed required a double extraction with acetonitrile, a precipitation and then LLE extraction with hexane–dichloromethane mixtures. In their method, it was necessary to use 300 µl of biological material. The protocol presented in the manuscript allows for the same LOQ as in the work of Eriksen et al. (2002); however, it is enough to extract only 200 μL volume samples. Thanks to the performed optimization, this volume was lowered from 1 mL that was reported by Péhourcq (2004), Kim et al. (2005) and Fortes et al. (2013) in their works.

The undoubted advantages of the presented method are LOQ of 5.0 µg/L and a small volume of biological material needed for extraction. These are the features that, combined with a very simple and fast method of sample preparation, determine a wide application possibilities of the presented method, including both clinical toxicology and forensic analysis.

### The application of the elaborated methodology in the forensic cases

The proposed analytical methods were successfully applied to a toxicological study. The fully validated method was applied to 28 authentic biological fluids (blood, urine, vitreous humor, bile and stomach content) requiring confirmation for HZ and CZ. The determination of HZ and CZ in vitreous humor, bile and stomach content was performed on urine and blood calibration curves, respectively. The analysis concerned 17 men aged 24 to 72 and 11 women aged 35 to 62. All samples were collected from either: traffic accident (thirteen cases), circulatory and respiratory failure (five cases), multiple drug poisoning (two cases), suicide (one case), death in a psychiatric hospital (one case), sepsis (one case), no clear cause of death (one case), fall from height (one case), lesions in the form of widely developed purulent peritonitis causing respiratory arrest (one case).

Antemortem blood concentrations in one cases (case number 15), CZ concentration was 187.1 ng/mL, but in two cases, antemortem blood (case number 2 and 15) and HZ concentrations were 8.9 ng/mL and 11.2 ng/mL, respectively. In postmortem blood, CZ has been found in concentrations ranging from 4.9 ng/ml to 596.5 ng/ml, and HZ at concentrations ranging from 6.0 ng/ml to 622.0 ng/ml. In different postmortem specimens, concentrations of CZ ranged within 5.9–390.6 ng/mL, 685.4–58,793.5 ng/mL, and 73.1 ng/mL in vitreous humor, urine, and bile, respectively. The concentration of HZ ranged within 4.6–65.7 ng/mL, 61.9–1700 ng/mL, and 47.9 ng/mL in vitreous humor, urine, and bile, respectively. Detailed information regarding determined substances, their concentrations as well as other toxicological findings are gathered in Table 6. In five cases (case number: 6, 7, 9, and 14), toxic level of HZ was found (TIAFT reference blood level list of therapeutic and toxic substances, September 2004. https://www.yumpu.com/en/document/view/13423269/tiaft-reference-blood-level-list-of-therapeutic-and-toxic-gtfch). No subjects died due to the fatal poisoning of CZ or HZ. In six of the cases (case 8, 9, 10, 13, 16, and 22), HZ and CZ were determined only in the blood from the victims or perpetrators of traffic accidents. In 25 of the cases, other xenobiotics were detected including antipsychotics, antidepressants, benzodiazepines, hypnotics and sedatives, and painkillers. Detailed information regarding determined substances, their concentrations as well as other toxicological findings are gathered in Table 6.

**Table 6:** Concentration of HZ and CZ in 28 authentic forensic cases with other toxicological findings with method of quantification of other xenobiotics with instrumental parameters

Case number Sex/age	Cause of death/clinical picture	CZ concentration in blood [ng/ml]	CZ concentration in other biological material [ng/ml]	HZ concentration in blood [ng/ml]	HZ concentration in other biological material [ng/ml]	Other xenobiotics concentration in blood [ng/ml]	Other xenobiotics concentration in other biological material [ng/ml]	Deutered internal standards (IS)	Sample pre-treatment	Method	Ion source	Acquisition mode	Column
Method 1													
Case 1 Male/50	Corpse found in home, circulatory and respiratory failure	29.2	VH: 29.1	8.2	VH: 10.9	Risperidone: 102.0 Paliperidone: 28.7	VH: Risperidone: 382.0 Paliperidone: 145.4	Amphetamine-D11 and diazepam-D5	LLE with ethyl acetate	LC–QqQ-MS/MS	APCI	d-MRM	Poroshell 120 EC-C18 (3.0 × 50 mm × 2.7 μm)
Case 2 Female/35	Antemortem	–	–	8.9	–	7-Aminoclonazepam: 11.5 Clonazepam: 5.3 Zolpidem: 7.0 Oxazepam: 13.2 Nordiazepam: 99.7 Temazepam: 5.8 Diazepam: 28.6		Amphetamine-D11 and diazepam-D5	LLE with ethyl acetate	LC–QqQ-MS/MS	APCI	d-MRM	Poroshell 120 EC-C18 (3.0 × 50 mm × 2.7 μm)
Case 3 Male/26	Fall from height, extensive fracturing of skull bones and significant brain damage	28.8	–	36.9	–	Lidocaine: 2550.2	–	Amphetamine-D11 and diazepam-D5	LLE with ethyl acetate	LC–QqQ-MS/MS	APCI	d-MRM	Poroshell 120 EC-C18 (3.0 × 50 mm × 2.7 μm)
Case 4 Female/41	Corpse found in home, circulatory and respiratory failure	18.8	–	51.3	–	Clindamycin: 61 Paracetamol: 1938	–	Amphetamine-D11 and diazepam-D5	LLE with ethyl acetate	LC–QqQ-MS/MS	APCI	d-MRM	Poroshell 120 EC-C18 (3.0 × 50 mm × 2.7 μm)
Case 5 Female/37	Corpse found in river backwater, no clear cause of death	–	–	–	U: 1700*	–	U: Carbamazepine: 28,600*	Amphetamine-D11 and diazepam-D5	LLE with ethyl acetate	LC–QqQ-MS/MS	APCI	d-MRM	Poroshell 120 EC-C18 (3.0 × 50 mm × 2.7 μm)
Case 6 Male/45	Suicide	–	–	120.0	U: 105.2	Alprazolam: 1.0 mCPP: 0.00 Trazodone: 9.0	U: Alprazolam: 1.0 mCPP: 1.0 Trazodone: 7.6	Amphetamine-D11 and diazepam-D5	LLE with ethyl acetate	LC–QqQ-MS/MS	APCI	d-MRM	Poroshell 120 EC-C18 (3.0 × 50 mm × 2.7 μm)
Case 7 Male/64	Corpse found in home, circulatory and respiratory failure	596.5	–	333.5	–	Quetiapine: 148.9	–	Amphetamine-D11 and diazepam-D5	LLE with ethyl acetate	LC–QqQ-MS/MS	APCI	d-MRM	Poroshell 120 EC-C18 (3.0 × 50 mm × 2.7 μm)
Case 8 Male/55	Traffic accident	37.4	–	54.8	–	–	–	Amphetamine-D11 and diazepam-D5	LLE with ethyl acetate	LC–QqQ-MS/MS	APCI	d-MRM	Poroshell 120 EC-C18 (3.0 × 50 mm × 2.7 μm)
Case 9 Female/47	Traffic accident	110.9	–	357.0	–	Diazepam: 61,4 Temazepam: 0,2	–	Amphetamine-D11 and diazepam-D5	LLE with ethyl acetate	LC–QqQ-MS/MS	APCI	d-MRM	Poroshell 120 EC-C18 (3.0 × 50 mm × 2.7 μm)
Case 10 Male/65	Traffic accident	61.,7	–	47.8	–	Pethidine: 23,2		Amphetamine-D11 and diazepam-D5	LLE with ethyl acetate	LC–QqQ-MS/MS	APCI	d-MRM	Poroshell 120 EC-C18 (3.0 × 50 mm × 2.7 μm)
Case 11 Male/65	Corpse found in home, circulatory and respiratory failure	84.4	U: 58,793.5	42.4	U: 64.9	–	–	Amphetamine-D11 and diazepam-D5	LLE with ethyl acetate	LC–QqQ-MS/MS	APCI	d-MRM	Poroshell 120 EC-C18 (3.0 × 50 mm × 2.7 μm)
Case 12 Male/72	Corpse found in home, circulatory and respiratory failure	37.4	–	54.9	–	–	–	Amphetamine-D11 and diazepam-D5	LLE with ethyl acetate	LC–QqQ-MS/MS	APCI	d-MRM	Poroshell 120 EC-C18 (3.0 × 50 mm × 2.7 μm)
Case 13 Male/61	Traffic accident	38.9	–	37.8	–	Lidocaine: 2460.4		Amphetamine-D11 and diazepam-D5	LLE with ethyl acetate	LC–QqQ-MS/MS	APCI	d-MRM	Poroshell 120 EC-C18 (3.0 × 50 mm × 2.7 μm)
Method 2													
Case 1 Male/50	Death in a psychiatric hospital	–	–	622.0	–	Quetiapine: 171.6 7-Hydroxy quetiapine: 1.1 Diazepam: 75.9 Nordiazepam: 35.2 Temazepam: 12.3 Oxazepam: 8.5 Norquetiapine ** o-Desalkylquetiapine** Metformin: 113.5 mCPP: 124.0 Trazodone: 187.8 Tamsulosin** Paracetamol** Dexamethasone**	–	Amphetamine-D11 and diazepam-D5	LLE with ethyl acetate	LC–QqQ-MS/MS	ESI	MRM	Acqutity UPLC® BEH C18 (2.1 × 50 mm × 1.7 µm)
Case 2 Female/34	Antemortem	187.1	–	11.2	–	Amphetamine: 24.7	–	Amphetamine-D11 and diazepam-D5	LLE with ethyl acetate	LC–QqQ-MS/MS	ESI	MRM	Acqutity UPLC® BEH C18 (2.1 × 50 mm × 1.7 µm)
Case 3 Male/45	Traffic accident	137.9	–	74.6	–	7-Aminoclonazepam: 21.0 Normorphine: 9.4 Morphine: 504.5 Codeine: 8.8 Papaverine: 0.8 Paracetamol: 133.5 7-Hydroxy quetiapine: 0.5 Quetiapine: 1.2 Haloperidol: 1.1	–	Amphetamine-D11 and diazepam-D5	LLE with ethyl acetate	LC–QqQ-MS/MS	ESI	MRM	Acqutity UPLC® BEH C18 (2.1 × 50 mm × 1.7 µm)
Case 4 Male/24	Drug addict, multiple drug poisoning	163.3	U: > 10,000 VH: 208.2	104.2	U: 287.5 VH: 65.7	Morphine: 17.6 Fentanyl: 22.5 7-Aminoclonazepam: 6.8 Alprazolam: 38.6 Nordiazepam: 11.0 Diazepam: 2.4 Sertraline: 6.3	U: Morphine: 2420.5 Normorphine: 217 Fentanyl: 254 7-Aminoclonazepam: 55 Alprazolam: 40 Nordiazepam: 2.6 Diazepam: 0.6 Sertraline: 1.3 cis-Tramadol: 8.3 Oxymorphone: 4.5 VH: Morphine: 46.8 Fentanyl: 28.6 7-Aminoclonazepam: 2.5 Alprazolam: 18.6 Nordiazepam: 1.9 Diazepam: 0.5 Sertraline: 3 cis-Tramadol: 2.8	Amphetamine-D11 and diazepam-D5	LLE with ethyl acetate	LC–QqQ-MS/MS-	ESI	MRM	Acqutity UPLC® BEH C18 (2.1 × 50 mm × 1.7 µm)
Case 5 Female/54	Lesions in the form of widely developed purulent peritonitis causing respiratory arrest	91.2	–	27.9	–	o-Desmethyltramadol: 209.3 cis-Tramadol: 1246 n-Desmethyltramadol: 978.4 Zolpidem: 50.4 Papaverine: 141.2 Drotaverine: 430.3 Paracetamol: 992.6 4-MAA: 235.9	–	Amphetamine-D11 and diazepam-D5	LLE with ethyl acetate	LC–QqQ-MS/MS	ESI	MRM	Acqutity UPLC® BEH C18 (2.1 × 50 mm × 1.7 µm)
Case 6 Male/26	Primary cause of death —autoimmune adipose tissue inflammation; secondary—sepsis, choking with blood	82.6	VH: 390.6 B: 73.1	22	VH: 19.2 B: 47.9	Lidocaine: 225.0 Metoprolol: 52.0 Fentanyl: 2.2 Levofloxacin and omeprazole metabolites**	VH: Lidocaine: 262 Metoprolol: 48.9 Fentanyl: 4.4 Levofloxacin and omeprazole metabolites** B: Lidocaine: 417.5 Metoprolol: 87.4 Fentanyl: 5.9 Levofloxacin quality and omeprazole metabolites**	Amphetamine-D11 and diazepam-D5	LLE with ethyl acetate	LC–QqQ-MS/MS	ESI	MRM	Acqutity UPLC® BEH C18 (2.1 × 50 mm × 1.7 µm)
Case 7 Female/62	Multidrug poisoning	271.5	U: > 10,000	SC: 85	SC: > 10,000	Paracetamol: 123 Lamotrigine: 4092 Pseudoephedrine: 3350 Amitriptyline: 956 Nortriptyline: 85 Estazolam: 215 Fluoxetine: 387.5 Chlorprothixene: 842	SC: Paracetamol, Lamotrigine, Pseudoephedrine, Amitriptyline, Estazolam, Fluoxetine, and chlorprothixene > 10,000	Amphetamine-D11 and diazepam-D5	LLE with ethyl acetate	LC–QqQ-MS/MS	ESI	MRM	Acqutity UPLC® BEH C18 (2.1 × 50 mm × 1.7 µm)
Case 8 Male/43	Traffic accident	4.9	VH: 5.9	–	–	o-Desmethyltramadol: 25.9 cis-Tramadol: 279.3 n-Desmethyltramadol: 27.5 Verapamil: ** Norverapamil: ** Telmisartan: **	VH: o-Desmethyltramadol: 35.6 cis-Tramadol: 307.9 n-Desmethyltramadol: 28.1 Verapamil: ** Norverapamil: ** Telmisartan: **	Amphetamine-D11 and diazepam-D5	LLE with ethyl acetate	LC–QqQ-MS/MS	ESI	MRM	Acqutity UPLC® BEH C18 (2.1 × 50 mm × 1.7 µm)
Case 9 Male/34	Traffic accident	112.4	–	10.5	–	Oxazepam: 8.9 Nordiazepam: 181.9 Temazepam: 3.3 Diazepam: 62.8 Quetiapine: 0.9 Sertraline: 1.1 Omeprazole and metabolites: ** Valproic acid: 17.8	-	Amphetamine-D11 and diazepam-D5	LLE with ethyl acetate	LC–QqQ-MS/MS	ESI	MRM	Acqutity UPLC® BEH C18 (2.1 × 50 mm × 1.7 µm)
Case 10 Male/63	Traffic accident	48.3	U: 685.4	27.9	U: 227.2	Desmethylcitalopram: 76.8 Escitalopram: 405.5 Amlodipine: 35.8 Paracetamol: 530.1	U: Desmethylcitalopram: 1323.4 Escitalopram: 7485.1 Amlodipine: 402.7 Paracetamol: 3929.4	Amphetamine-D11 and diazepam-D5	LLE with ethyl acetate	LC–QqQ-MS/MS	ESI	MRM	Acqutity UPLC® BEH C18 (2.1 × 50 mm × 1.7 µm)
Case 11 Male/51	Traffic accident	236.9	U: > 10,000	37.7	U: 905.8	7-Aminoclonazepam: 470.5 Codeine: 1.9 Morphine: 933.1 Normorphine: 19.9 Amphetamine: 11.6 THC: 2.8 THC-COOH: 23.2 Naloxone: ** Amiodarone: 7582.2 Atropine: 384.1	U: Clonazepam: 18.5 7-Aminoclonazepam: 2421.3 Codeine: 138.3 Morphine: > 10 000 Normorphine: 1583.1 Amphetamine: 2544.4 THC-COOH: 24.0 Naloxone: ** Amiodarone: 67.1 Atropine:2.2 Dextrorphan: 1.0	Amphetamine-D11 and diazepam-D5	LLE with ethyl acetate	LC–QqQ-MS/MS	ESI	MRM	Acqutity UPLC® BEH C18 (2.1 × 50 mm × 1.7 µm)
Case 12 Female/43	Traffic accident	134.3	VH: 118.6	31.0	VH: 6.8	Paracetamol: 203.5 Propranolol: 18.5 Lidocaine: 35.2	VH: Paracetamol: 96.7 Propranolol: 19.4 Lidocaine: 43.0	Amphetamine-D11 and diazepam-D5	LLE with ethyl acetate	LC–QqQ-MS/MS	ESI	MRM	Acqutity UPLC® BEH C18 (2.1 × 50 mm × 1.7 µm)
Case 13 Female/38	Traffic accident	36.2	U: 1097.2	43.1	U: 61.9	7-Aminoclonazepam: 870.1 Oxycodone: 3.6 Oxymorphone: 2.4 Morphine: > 1000 Normorphine: 49.2 Mianserin: 52.7 Escitalopram: 80.5 Desmethylcitalopram: 250.1	U: 7-Aminoclonazepam: 6058.6 Oxycodone: 5.1 Oxymorphone: 9.9 Morphine: > 1000 Normorphine: 12.0 Mianserin: 250.6 Escitalopram: 264.4 Desmethylcitalopram: 1871.2	Amphetamine-D11 and diazepam-D5	LLE with ethyl acetate	LC–QqQ-MS/MS	ESI	MRM	Acqutity UPLC® BEH C18 (2.1 × 50 mm × 1.7 µm)
Case 14 Female/42	Traffic accident	43.2	U: 2365.9	36.2	U: 63.6	Diazepam: 1.8 Fentanyl: 0.6 Olanzapine: 30.7 Atropine: 466.9 Lidocaine: 1.6 Metoclopramide: 127.2 Metoprolol: 36.0 Mianserin: 22.7 Tianeptine: 0.0 Etomidate: 30.8	U: Nordiazepam: 5.7 Fentanyl: 0.2 Olanzapine: 81.5 Atropine: 33.9 Lidocaine: 6.3 Metoclopramide: 7940.2 Metoprolol: 685.0 Mianserin: 27.2 Tianeptine: 1.5 Etomidate: 2.5	Amphetamine-D11 and diazepam-D5	LLE with ethyl acetate	LC–QqQ-MS/MS	ESI	MRM	Acqutity UPLC® BEH C18 (2.1 × 50 mm × 1.7 µm)
Case 15 Female/44	Traffic accident	6.3	VH: 10.4	6.0	VH: 4.6	Clozapine: 4770.4 Norclozapine: ** Clozapine n-oxide: ** Paliperidone: 76.0	VH: Clozapine: 883.5 Norclozapine: ** Clozapine n-oxide: ** Paliperidone: 361.5	Amphetamine-D11 and diazepam-D5	LLE with ethyl acetate	LC–QqQ-MS/MS	ESI	MRM	Acqutity UPLC® BEH C18 (2.1 × 50 mm × 1.7 µm)

*[ng/g]; **qualitative confirmation

*4-MAA* 4-methylaminoantipyrine, *B* bile, *CZ* cetirizine, *HZ* hydroxyzine, *SC* stomach content, *THC* Δ^9^-tetrahydrocannabinol, *THC-COOH* 11-nor-9-carboxy-Δ^9^-tetrahydrocannnabinol, *U* urine, *VH* vitreous humor, *mCPP* 1-(3-chlorophenyl)piperazine, *LLE* liquid–liquid extraction, *ESI* electrospray ionization, *APCI* atmospheric-pressure chemical ionization, *MRM* Multiple Reaction Monitoring, *d-MRM* dynamic Multiple Reaction Monitoring

Blood HZ concentration exhibited a median of 100 µg/L (range 60–370 µg/L) in 34 person arrested for impaired driving and their averaged 170 µg/L in an additional 5 person (Baselt 2017). In three people whose death was attributed solely to an acute overdose of HZ, its blood concentration was found at the following levels: 0.7, 2.5, and 3 µg/ml. In the case of an 18-year-old woman who died as a result of an overdose of HZ, its concentration in blood and urine was 4.2 and 1.4 µg/ml, respectively (Baselt 2017). The analysis using the developed UHPLC–QqQ-MS/MS method performed on blood, urine, vitreous, and bile samples showed that the HZ concentration was within this range, but lower levels were also observed. It is worth adding that in many of the presented cases, other substances were detected in the biological material, which could have influenced the general condition of the deceased. For example, drugs that depress the central nervous system, i.e., sedatives, hypnotics, anesthetics, benzodiazepines or opioids, act similarly to HZ, which acts synergistically with agents that depress the CNS, thereby intensifying the effects of the latter.

## Conclusion

Various techniques suitable for the detection and determination of HZ and CZ in biological samples have been described so far. Initially, the liquid chromatography technique with spectrophotometric (UV) and fluorescence (FL) detection were used. These methods were characterized by low sensitivity and selectivity. It was, therefore, necessary to develop a method using liquid chromatography and gas chromatography combined with mass spectrometry, which is characterized by high sensitivity, specificity, selectivity and which enables the simultaneous detection and determination (in the tested materials) of various (and several) substances in low concentrations in the biological samples. The application of high-resolution mass spectrometry enables unambiguous identification of HZ and CZ in postmortem whole blood. The developed efficient method is characterized by high recovery rate, low cost, and fast sample preparation. The accuracy, reproducibility, simplicity, and selectivity of the elaborated chromatographic method suggest its application in clinical, toxicological, and forensic laboratories, which was already confirmed by the studies performed on the toxicological samples that were presented in the manuscript above.

## Data Availability

The data that support the findings of this study are available from the corresponding author upon reasonable request.
